# Inhibition of return for body images in individuals with shape/weight based self-worth

**DOI:** 10.1186/s40337-018-0211-5

**Published:** 2018-09-14

**Authors:** Alexandra Cobb, Elizabeth Rieger, Jason Bell

**Affiliations:** 1 Research School of Psychology, Australian National University, Canberra, ACT 2601 Australia; 2 Research School of Psychology, Australian National University, Canberra, ACT Australia; 3 School of Psychological Science, University of Western Australia, Perth, WA Australia

**Keywords:** Eating disorder, Attention, Inhibition of return, IOR, Shape/weight concerns

## Abstract

**Background:**

Attentional biases for body shape and weight information have been found in people with eating disorders, indicating disorder-specific changes in the way this information is processed. To date, the literature has focused on the initial capture of attention, with little research on the maintenance of attention to shape/weight-related information. The current study aims to investigate the occurrence of attentional maintenance through the use of an Inhibition of Return task to shape and weight stimuli in those with and without an eating disorder.

**Method:**

Three groups of female participants between the ages of 16–30 years undertook an Inhibition of Return task with target images of female bodies and control images of animals. The groups were an eating disorder group (*n* = 20), a High shape/weight based self-worth group (*n* = 23), and a Low shape/weight based self-worth group (*n* = 26).

**Results:**

The results indicated differential patterns of Inhibition of Return between the High and Low shape/weight based self-worth groups. The High group displayed increased inhibition of return for the shape/weight stimuli relative to control stimuli, while the Low group displayed reduced inhibition of return for the shape/weight stimuli compared to control stimuli. The ED group displayed a similar pattern of results to the High group, but this did not reach significance.

**Conclusion:**

The current findings indicate that young women without an eating disorder who base their self-worth on shape/weight display a pattern of avoidance of shape/weight stimuli that is in direct contrast to those at low risk of developing eating disorders. The possible implications of these specific patterns of inhibition of return across those at varying levels of risk for an eating disorder are discussed along with their implications for intervention approaches.

**Electronic supplementary material:**

The online version of this article (10.1186/s40337-018-0211-5) contains supplementary material, which is available to authorized users.

## Plain English summary

Research has indicated differences in the way body shape and weight information captures the attention of those with an eating disorder versus those without an eating disorder. Despite this, it is unclear whether attentional differences persist after this initial capture. The current research aims to answer this question by comparing the later stages of attention in a control group, a group at high risk of an eating disorder and a clinical eating disorder group. All three groups performed an attentional inhibition of return task. Results indicate that those at risk of an eating disorder avoid maintaining their attention on body shape and weight information.

## Background

Eating disorders are complex mental and physical illnesses that centre on disturbances in how body shape and weight are experienced and evaluated [1]. Several models posit that these disorders entail maladaptive knowledge structures that are involved in the allocation of attention [2, 3]. Vitousek and Hollon’s cognitive model [4] proposes that those with eating disorders develop extensive and inaccurate schemas regarding their body shape and weight. These faulty schemas lead to automatic thoughts and systematic errors in thinking, such as selective attention and confirmation biases towards weight/shape information [5]. Aspen et al. [5] extended this approach and posited that attentional biases are a strong contributor to the development and maintenance of eating disorders.

Supporting this conceptualisation, a large body of research has indicated that those with eating disorders exhibit attentional biases regarding disorder-salient information. Attentional biases can be conceptualised to include three components of attention: facilitated attentional capture, difficulty in disengagement, and attentional avoidance [6]. In the context of clinical disorders, facilitated attention refers to the finding that disorder salient information is often detected faster than non-salient information. Difficulty in disengagement refers to disorder salient information being harder to disengage from relative to non-salient information. Attentional avoidance refers to attention being directed away from disorder salient information. Each of these three components of attentional biases can be investigated using various attention tasks.

A large body of research has investigated the occurrence of facilitated attention and disengagement in eating disorders. Meta-analyses on the cognitive paradigms of the Stroop task and dot probe paradigm have consistently shown attentional biases regarding eating disorder-salient stimuli, such as shape/weight related words, in women diagnosed with an eating disorder [5, 7, 8]. While the Stroop task has been widely used to investigate attentional biases in eating disorders, it has a number of limitations that restrict its utility, including an inability to differentiate between the allocation of attention towards or away from target stimuli [9].

Accordingly, researchers have employed alternative paradigms such as a modified dot-probe procedure and eye tracking. Aspen et al. [5] conducted a meta-analysis on the dot-probe paradigm and concluded that those with eating disorders display an attentional bias towards stimuli connoting a larger physique, and away from stimuli connoting a thin physique. However, some research demonstrates the reverse, with vulnerable individuals (defined as those with a high level of shape/weight dissatisfaction or those endorsing eating disorder symptomatology) displaying an attentional bias towards thin stimuli and away from non-thin stimuli [10–14]. Eye tracking studies have also shown inconsistent findings, with some research indicating an attentional bias towards negatively perceived body parts [13, 15, 16] and others indicating an avoidance response [17]. While the conditions specifying the occurrence of facilitation versus avoidance of shape and weight information remain to be specified, their occurrence in individuals with eating disorder symptoms has been established.

One limitation of this research is its predominant focus on the initial stages of attention [18]. However, attention is enduring, and difficulty in disengaging attention or ongoing attentional avoidance may therefore have a role in the continuation of many disorders. For eating disorders, understanding difficulties in attentional disengagement is important, given the high level of preoccupation with shape and weight concerns [19, 20].

One way to investigate attentional disengagement is through the Inhibition of Return (IOR) phenomenon, measured using an attentional cuing task [21]. IOR has traditionally been defined as a mechanism of the visual system that encourages attention towards novel locations by inhibiting attention from returning to previously attended locations [22]. More recent work (particularly within clinical fields of research) has compared the magnitude of IOR to different types of stimuli in order to examine differences in attentional disengagement. The critical comparisons then become: 1) does IOR occur for that stimulus type, and 2) does IOR magnitude differ by stimulus type. The disengagement hypothesis of attention suggests that the process of attending includes initial capture, followed by attentional disengagement [6, 23]. A reduced IOR to one type of image but not another indicates a reduced ability to disengage attention from certain information (e.g., shape/weight stimuli). In contrast, an increased IOR to a specific image type indicates increased ability to disengage from that stimulus or a potential avoidance response.

Attentional cueing tasks have been used to research the occurrence of attentional disengagement in depressed and anxious populations [23, 24]. Results of these studies not only show attentional facilitation biases, but also increased difficulty in attentional disengagement from threatening and negative stimuli. Thus, it may be the combination of attentional capture and attentional disengagement difficulties that influences the occurrence and maintenance of these disorders.

**Fig. 1:**
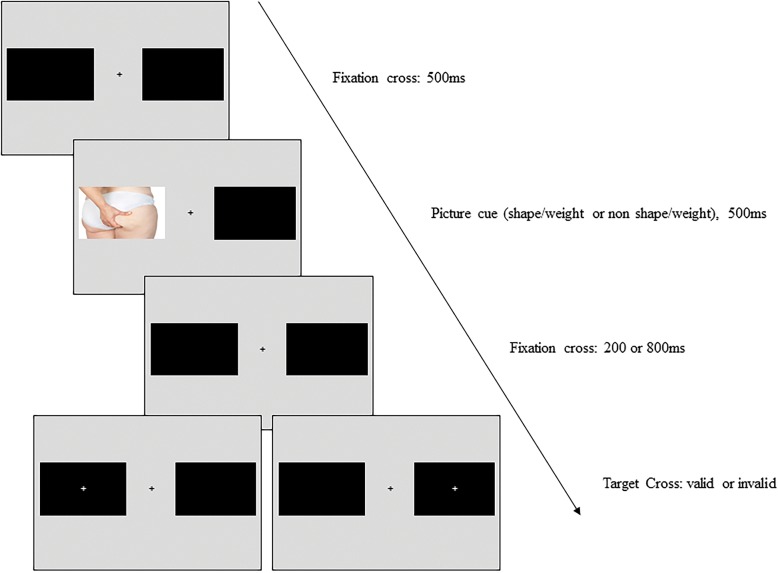
IOR trial sequence

An important consideration in the investigation of attentional disengagement is any change in the time course of disengagement, which may vary across participant groups [23]. As depicted in Fig. 1, one way to investigate disengagement is through the use of differing times between the cue (e.g. a shape/weight image) and the target (e.g., a valid or invalid located cross) onset times, known as the stimulus onset asynchrony (SOA). As outlined by Klein [22], at short SOA times, facilitation occurs, indicated by faster response times to valid target than an invalid target. However, as the SOA times increase, facilitation transitions into an inhibition effect as indicated by faster response times to invalid target than a valid target. The inhibition mechanism typically occurs from 300 ms to 1500 ms [22].

To date, only one study has investigated attentional disengagement in the context of body dissatisfaction [11]. Using an undergraduate female Chinese sample, this study found that those who experienced higher levels of body weight dissatisfaction had greater difficulty disengaging their attention from shape and weight images (i.e., a smaller IOR effect relative to the control group). Another study investigated attentional maintenance in a sample of people in the obese and normal-weight ranges and found that individuals with obesity had greater difficulty disengaging their attention from food stimuli (i.e., a smaller IOR effect relative to the control group) [25]. These studies indicate the potential for food and shape information to not only capture attention, but to also lead to greater difficulty in attentional disengagement. Further, an eye-tracking study within a non-clinical population found that those with higher body dissatisfaction had difficulty disengaging their attention from their most dissatisfying body regions when compared with controls [26]. However, while eye-tracking studies provide information as to where eye gaze is directed, they do not provide a clear index regarding which stimuli are being processed (as, for example, IOR tasks provide). Despite its strengths, no research on attentional disengagement using an IOR paradigm has been conducted within an eating disorder population.

In addition to determining whether individuals with eating disorders display distorted attentional disengagement, it is also of interest to determine whether those processes extend to individuals at risk of eating disorders, such as those with high levels of shape and/or weight-based self-worth. Cognitive models postulate that a central feature of the cognitive dysfunction characterising eating disorders is the unification of shape and weight concerns with a person’s self-evaluation [19, 27, 28]. This shape/weight-based self-worth is thought to drive eating disorder behaviours to improve self-worth and is required for a *DSM-5* eating disorder diagnosis [1]. While no research has investigated the relationship between shape/weight-based self-worth and attentional disengagement, it is possible that shape/weight stimuli would have particular salience in those for whom shape/weight-related information has implications for self-worth, and therefore may result in altered attentional disengagement.

Using an attentional cueing task, the current study aims to investigate the occurrence and time course of attentional disengagement to shape/weight stimuli in the eating disorder context (including those with eating disorders and those at risk of an eating disorder due to elevated levels of shape/weight-based self-worth) at two differing SOA times. Given empirical and theoretical work suggesting that those with eating disorder symptoms experience biases in the initial stages of attention, and evidence of difficulties with attentional disengagement in other clinical populations, it is expected that those with an eating disorder and those with high shape/weight-based self-worth will experience greater difficulty disengaging their attention from shape/weight stimuli, relative to individuals with low shape/weight based self-worth, at the longer SOA time (i.e., when inhibited processing, or disengagement of attention, is typically expected to occur).

## Methods

### Participants

Fifty-eight female undergraduate university students aged between 16 and 30 years were recruited via the Australian National University’s online recruiting system. Twenty-one females between the ages of 16–30 years who were currently seeking treatment from a public outpatient eating disorders program were recruited via flyers. All participants provided informed consent prior to participating in the study. Those aged between 16 and 18 years also provided parental consent. Ethical approval was granted by the ACT Health Human Research Ethics Committee (ETH.5.14.110) and the Australian National University Human Research Ethics Committee (2014/448).

The current study included three groups of participants: an eating disorder (ED) group, a high shape/weight concern (High) group, and a low shape/weight concern (Low) group. All participants were required to be female, given that images of females were used in the attentional task. All participants were also required to have a BMI below 25 kg/m^2^, as research has indicated that the experience of being overweight or obese can have significant effects on attentional biases [29], including attentional maintenance [25]. Ten participants were excluded on this basis. Participants’ BMIs ranged from (16.38–25.00).

To be included in the ED group, participants were required to have a current *DSM-5* [1] eating disorder as diagnosed by a clinician experienced in eating disorders. In line with a transdiagnostic model [19], no distinction was made between those with differing eating disorder diagnoses. The ED group was comprised of seven participants with a diagnosis of anorexia nervosa, six with a diagnosis of bulimia nervosa, and seven with a diagnosis of other specified feeding or eating disorder (OSFED).

Participants were excluded from the two non-eating disorder groups if they had symptoms suggestive of an eating disorder, as indicated by a BMI below 17.0 and/or endorsement of the eating disorder behaviours (items 13–18) assessed by the Eating Disorders Examination Questionnaire (EDE-Q) [30] at a level that may indicate a *DSM-5* eating disorder diagnosis. One participant was excluded on this basis. To be included in the High group, participants were required to score 4 or above on item 22 (‘Has your weight influenced how you think about [judge] yourself as a person?’) or item 23 (‘Has your shape influenced how you think about [judge] yourself as a person?’) of the EDE-Q, indicating moderate or greater than moderate investment in their shape/weight as a basis for self-worth. Those who scored three or below on items 22 and 23 were included in the Low group, indicating below moderate investment in their shape/weight as a basis for self-worth.

The final sample consisted of 20 participants in the ED group, 23 participants in the High group, and 26 participants in the Low group. The sample primarily consisted of Caucasian females born in Australia who had obtained senior secondary education. Table 1 depicts the ethnic and educational characteristics of the sample.

**Table 1:** Country of origin and educational attainment across groups

	Low	High	ED
Country of Origin	Percentage		
Australia	30.8	33.3	80.0
New Zealand	3.8	0	0
China	15.4	37.5	0
Malaysia	15.4	4.2	5.0
Singapore	3.8	0	0
Other Asian Country	26.9	16.7	5.0
Other English Speaking Country	0	8.3	5.0
Other non-English Speaking Country	3.8	0	5.0
Educational Attainment	Percentage		
Pre-Primary	0	0	0
Primary	0	0	0
Junior Secondary	0	0	25
Senior Secondary	65.4	75.0	50
Certificate Level	7.7	0	0
Advanced Diploma and Diploma Level	0	4.2	0
Bachelor Degree	19.2	20.8	10
Graduate Diploma/Graduate Certificate	3.8	0	0
Postgraduate Degree	3.8	0	5

Junior Secondary = Year 10 or equivalent, Senior Secondary = Year 12, senior secondary certificate or equivalent. Low = Low shape/weight based self-worth group, High = High shape/weight based self-worth group, ED = Eating Disorder group

### Materials and measures

#### IOR task

The attentional cueing IOR task was administered on a laptop computer using Matlab R2012b software. It contained 22 shape/weight images and 22 animal images, which were sourced from photographic libraries or non-copyrighted images on the internet. A pilot study (unpublished) was conducted using a large pool of target (shape/weight) and control (animal) images. The target image set was designed to contain images of female body parts within the normal to overweight BMI range (consistent with the normative BMI range for Australian women) [31] with a focus on body parts associated with shape/weight concerns (i.e., stomach, thighs, and buttocks). Consistent with the method employed by the International Affective Picture System [32], the initial pool of shape and animal images was rated for valence (i.e. “how happy or unhappy the images make you feel”) and arousal (i.e. “how exciting or calm the images make you feel”) on 9-point Likert Self-Assessment Manikins scale, by a separate sample of female undergraduate university students (*n* = 42). Twenty two shape/weight and 22 animal images were selected from this pool based on matched ratings on valence and arousal to ensure that any differences in attention were not the result of differences in the valence or arousal of the image sets. Pilot data indicated no significant difference in the ratings of valence, *t*(22) = − 1.11, *p* = .276, between the shape/weight image set (*M* = 4.01, *SD* = .70) and animal image set (*M* = 4.31, *SD* = 1.06), or arousal, *t*(22) = − 1.17, *p* = .25, between the shape/weight image set (*M* = 4.38, *SD* = 0.31) and animal image set (*M* = 4.65, *SD* = 1.04).

The IOR task was a spatial cueing task based on previous designs [21, 22]. Figure 1 depicts the sequence of a single trial, which consisted of a 500 ms presentation of a central grey fixation cross, which remained on the screen throughout the trial. Following this, a picture cue, either a shape/weight image or an animal image, was presented for 500 ms in a lateral position, either 3 degrees left or 3 degrees right of the fixation cross. This was followed by a change in brightness of the central fixation cross to cue participants’ attention back to the centre of the screen. Following this, a white target cross appeared in either the same location as the picture cue (valid trial) or in the opposite location (invalid trial). The time between cue picture onset and target cross appearance, the SOA time, was either 700 ms or 1300 ms [22]. Participants were instructed to respond using the left and right arrow keys on the laptop keyboard to indicate, as quickly and accurately as possible, on which side of the screen the target appeared. The next trial began after the participants’ response, or after 2000 ms.

To ensure the participants’ understanding, six practice trials were undertaken at the beginning of the task. Following this, participants undertook two blocks of 80 repetitions, comprising 10 repetitions of each of the eight conditions: 2 x picture type, 2 x picture location, and 2 x SOA. Twenty trials containing no target were randomly interspersed amongst the trials to ensure participant engagement and to prevent response rhythms. In total, participants completed 186 trials.

#### Eating disorder examination questionnaire 6.0 (EDE-Q) [33]

In order to assess the severity and frequency of eating disorder psychopathology, aid in the allocation to either the High and Low groups, screen for possible eating disorder diagnoses within the control groups, and ensure the validity of the eating disorder diagnoses in the clinical group, the EDE-Q was administered to all participants. The EDE-Q is a 28-item self-report version of the semi-structured interview based Eating Disorder Examination [34]. The EDE-Q contains four subscales of specific eating disorder psychopathology including Dietary Restraint (e.g., “Have you been deliberately trying to limit the amount of food you eat to.influence your shape or weight?”), Eating Concern (e.g., “Have you been afraid of losing control over eating?”), Weight Concern (e.g., “Have you had a strong desire to lose weight?”), and Shape Concern (e.g., “Have you felt fat?”), as well as a global eating disorder score.

The EDE-Q is widely used as it has sound psychometric properties, with research indicating high levels of convergent validity with the EDE interview [35], accuracy in differentiating those with and without eating disorders [36], and test-retest reliability [37, 38] and good internal consistency [36, 39]. In the current study, an acceptable level of internal consistency for the global EDE-Q scale was evidenced by the Cronbach’s alphas for the ED group (α = .90), the High group (α = .82), and the Low group (α = .86). The four subscales also indicated acceptable levels of internal consistency (given the small number of items in each scale), with a Cronbach’s alpha of .873 for the Restraint subscale, .859 for the Eating Concern subscale, .917 for the Shape Concern subscale, and .890 for the Weight Concern subscale.

#### Body mass index

Participants’ weight was recorded to the closest 0.1 kg and height measured to the closest centimetre for the calculation of BMI. The World Health Organization BMI classifications were used to classify groups as overweight (25 to 29.99) or obese (30 or greater) for screening purposes.

### Procedure

Testing sessions lasted approximately 45 min and were conducted in a quiet, private, and well-lit room. Upon arrival, participants completed a written consent form. They then undertook the IOR task and another IOR cognitive task for a separate study, in random order. Between these tasks, participants completed a distraction task aimed at reducing the risk of picture exposure in one task affecting the completion of the other task. The distraction task comprised watching 186 s of music film clips, obtained from the Database for Emotion Analysis [40]. The clips had been previously rated as neutral for valence and arousal, so were unlikely to induce any mood effects; further, they did not contain any eating disorder salient information (i.e., food or bodies) [40]. Following the IOR task, participants were administered the EDE-Q. At the completion of the session, participants in the Low and High groups had their height and weight measured using calibrated scales and a wall mounted tape measure (this information was obtained from the outpatient treatment facility for the ED group) and remuneration was provided.

### Statistical analysis

All data were analysed using IBM SPSS version 23. Prior to conducting the main analyses, data underwent a screening and cleaning process aimed at assessing the occurrence of missing data, outliers, and violations of statistical assumptions. The screening and cleaning process for the IOR data was conducted at both the individual and group levels. At the individual level, one case of missing data was identified and excluded listwise. Further, reaction times less than 200 ms or greater than 2.5 standard deviations above the mean were removed, as these scores were likely to indicate disengagement from the task, which is typical in tasks of this nature [11, 25, 41]. Accuracy data was screened, with all participants obtaining accuracy scores above 75%, indicating a high level of task compliance and engagement. IOR index scores were then calculated by subtracting the mean reaction time of the invalid trials from the mean reaction time of the valid trials. Positive IOR index scores indicated IOR, or a reluctance to return attention to a previously attended location. In contrast, negative IOR index scores indicated a facilitation effect or the absence of an IOR effect. A mixed between-within analysis of variance (ANOVA) was undertaken to analyse the IOR data. The dependant variable was the IOR indices, the between-subjects factor was group (Low, High, ED) and the within-subjects factors were picture type (shape/weight, animal) and SOA (1300 ms, 1700 ms). A two-tailed *p*-level of < 0.05 was used for determining statistical significance.

## Results

### Descriptive characteristics

To assess age and education across groups, a one-way between groups ANOVA was conducted. This indicated no significant difference in age, *F*(2, 67) = 2.31, *p* = .107, or years of education, *F*(2, 67) = .283, *p* = .755, between the groups. As expected, there were significant differences in eating disorder symptomatology between the three groups. Results indicated significant differences in the EDE-Q Dietary Restraint, (*F*(2, 47.80) = 17.62, *p* < .001, Eating Concern, *F*(2, 49.75) = 64.03, *p* < .001 (using the Brown-Forsyth adjustment), Shape Concern, *F*(2, 67) = 55.82, *p* < .001, Weight Concern, *F*(2, 67) = 49.80, *p* < .001 sub scales and Global scale, *F*(2, 67) = 65.01, *p* < .001. Post hoc comparisons using the Tukey HSD test indicated that all comparisons between the means of the ED, High, and Low groups were significantly different. The ED group displayed the highest global and subscale scores, followed by the High group endorsing a moderate level of ED pathology, and the low group endorsing the lowest global and subscale scores. The EDE-Q data thus supported the conceptualisation of the groups. Table 2 presents the mean age, BMI, and EDE-Q scale scores for each of the groups.

**Table 2:** Age, BMI and EDE-Q scores of the low shape/weight based self-worth, high shape/weight based self-worth, and eating disorder groups

	Low *n* = 26		High *n* = 23		ED *n* = 20	
	Mean	SD	Mean	SD	Mean	SD
Age	20.08	2.13	19.88	1.77	21.60	4.38
BMI	20.52	2.40	21.10	2.00	21.31	2.51
Global	1.04	.77	2.85	.94	4.29	1.21
Eating	.54	.60	2.09	1.17	4.05	1.21
Restraint	.89	.93	2.24	1.47	3.47	1.81
Shape	1.61	1.25	3.81	.93	5.03	1.16
Weight	1.12	.96	3.25	1.18	4.6	1.46

Global = EDE-Q Global scale score; Eating = EDE-Q Eating Concern subscale score; Restraint = EDE-Q Dietary Restraint subscale score; Shape = EDE-Q Shape Concern subscale score; Weight = EDE-Q Weight Concern subscale score

### Main analyses

Table 3 displays the mean reaction time data used to calculate the IOR index. A three-way, 2 (SOA: 700 ms and 1300 ms) × 2 (Image: Shape/Weight and Animal) × 3 (Group: ED, High, and Low), mixed design ANOVA revealed no significant main effect for image, Wilks’ Lambda = .974, *F*(1, 66) = 1.75, *p* = .191*, η*_*p*_^*2*^ = .026, SOA, Wilks’ Lambda = 1.00, *F*(1, 66) = .003, *p* = .955*, η*_*p*_^*2*^ < .001, or group, *F*(2, 66) = .439, *p* = .646*, η*_*p*_^*2*^ = .013. Further, there were no significant two-way interactions for image x group, Wilks’ Lambda = .985, *F*(2, 66) = .508, *p* = .604*, η*_*p*_^*2*^ = .015, SOA x group, Wilks’ Lambda = .985, *F*(2, 66) = .499, *p* = .610*, η*_*p*_^*2*^ = .015, or image x SOA, Wilks’ Lambda = .975, *F*(2, 66) = 1.71, *p* = .196*, η*_*p*_^*2*^ = .025. However, there was a significant three-way interaction between group, image, and SOA, Wilks’ Lambda = .889, *F*(2, 66) = 4.13, *p* = .020*, η*_*p*_^*2*^ = .111. To explore this interaction effect further, the data at each SOA was examined separately, as is typical for IOR experiments involving multiple SOAs [11, 24].

**Table 3:** IOR reaction time data for group, image type, and validity conditions (M ± SD, Milliseconds)

	Low Shape/Weight Based Self-Worth		
		700 SOA	1300 SOA
Shape/Weight	Valid	431.69 ± 45.31	404.42 ± 39.88
	Invalid	410.23 ± 37.80	397.69 ± 42.78
Animal	Valid	418.69 ± 42.01	409.95 ± 41.06
	Invalid	400.77 ± 36.72	391.00 ± 38.76
	High Shape/Weight Based Self-Worth		
		700 SOA	1300 SOA
Shape/Weight	Valid	460.35 ± 44.20	437.01 ± 46.99
	Invalid	447.22 ± 50.53	418.22 ± 44.61
Animal	Valid	467.09 ± 58.21	425.78 ± 41.53
	Invalid	441.69 ± 48.62	421.96 ± 39.27
	Eating Disorder		
		700 SOA	1300 SOA
Shape/Weight	Valid	467.90 ± 85.37	446.75 ± 71.30
	Invalid	459.70 ± 97.38	431.30 ± 85.83
Animal	Valid	464.45 ± 93.95	447.6 ± 90.77
	Invalid	452.45 ± 90.44	438.85 ± 90.35

Shape/Weight = Shape/Weight Images, Animal = Animal Images. Valid = Target in valid location, Invalid = Target in invalid location. SOA = Stimulus onset asynchrony time in milliseconds. Global = EDE-Q Global scale score; Eating = EDE-Q Eating Concern subscale score; Restraint = EDE-Q Dietary Restraint subscale score; Shape = EDE-Q Shape Concern subscale score; Weight = EDE-Q Weight Concern subscale score

#### 700 ms SOA

**Fig. 2:**
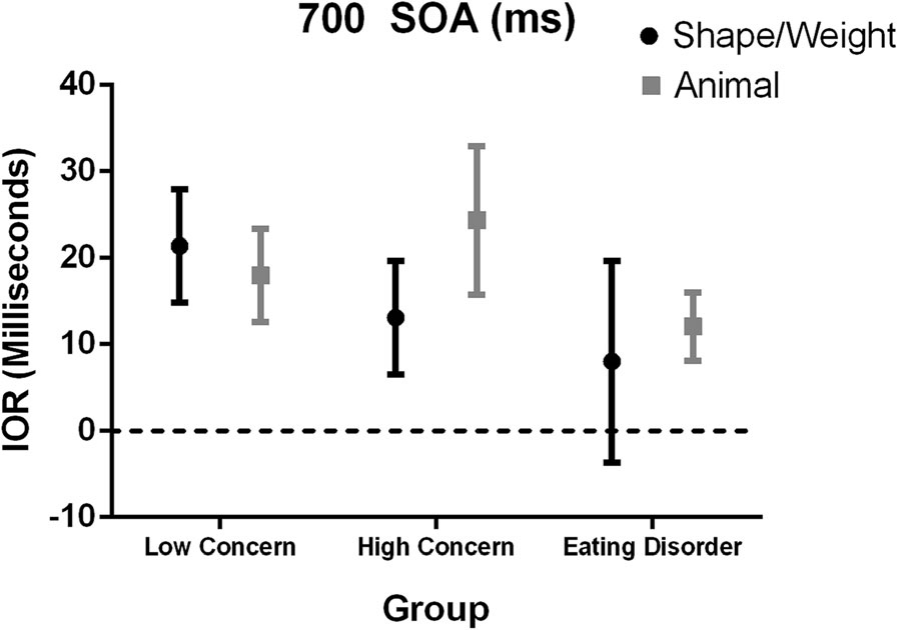
IOR Indices for the Low, High and ED groups for shape/weight and animal images at 700 ms SOA. Error bars +/− one standard error of the mean

A mixed design ANOVA revealed no significant main effect for image, Wilks’ Lambda = .991, *F*(1, 66) = .604, *p* = .440*, η*_*p*_^*2*^ = .009, or group, *F*(2, 66) = .760, *p* = .472*, η*_*p*_^*2*^ = .023. Further, there was no significant interaction between image and group, Wilks’ Lambda = .978, *F*(2, 66) = .744, *p* = .479*, η*_*p*_^*2*^ = .022, indicating that there was no significant difference in IOR for the shape/weight and animal images between the three groups. Figure 2 depicts the data for 700 ms SOA.

#### 1300 ms SOA

**Fig. 3:**
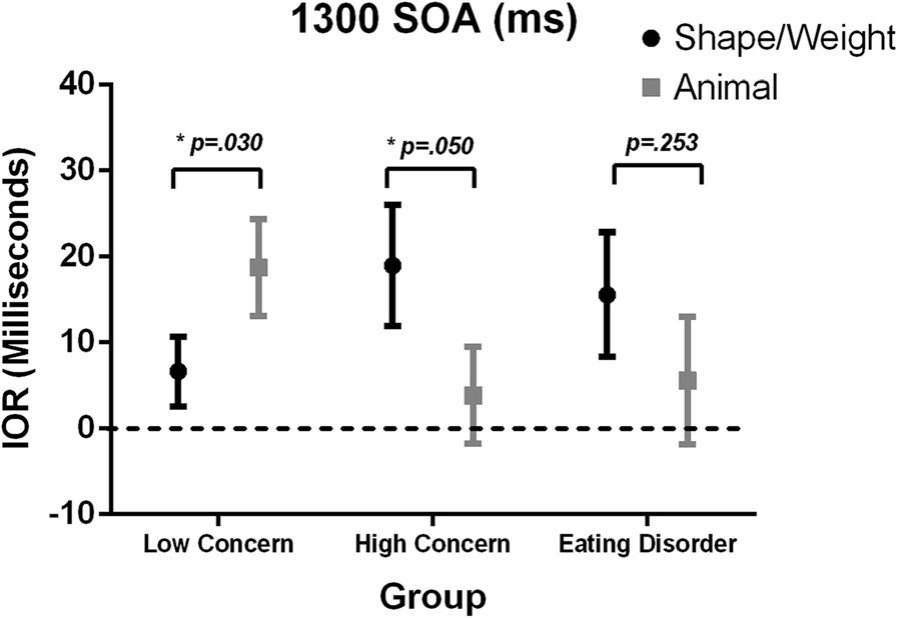
IOR Indices for Low, High and ED groups for shape/weight and animal images at 1300 ms SOA. Error bars +/− one standard error of the mean

A mixed design ANOVA revealed no significant main effects for image type, Wilks’ Lambda = .983, *F*(2, 66) = 1.16, *p* = .285*, η*_*p*_^*2*^ = .017, or group, *F*(2, 66) = .046, *p* = .955*, η*_*p*_^*2*^ = .001, but did reveal a significant interaction between image type and group, Wilks’ Lambda = .877, *F*(2, 66) = 4.64, *p* = .013*, η*_*p*_^*2*^ = .123, as shown in Fig. 3. To further clarify the nature of these results at the group level, paired samples *t*-tests were used to assess the difference in IOR for differing image types within the groups. The Low group exhibited significantly lower IOR, *t*(25) = − 2.26, *p* = .033, for the shape/weight images (*M* = 6.65, *SD* = 20.59) than the animal images (*M* = 18.73, *SD* = 28.70). By contrast, the High group displayed significantly higher IOR, *t*(22) = 2.07, *p* = .050, for the shape/weight images (*M* = 18.96, *SD* = 33.71) than the animal images (*M* = 3.87, *SD* = 26.89). The ED group also displayed higher IOR for the shape/weight images (*M* = 15.55, *SD* = 32.43) than the animal images (*M* = 5.55, *SD* = 33.17) but this trend was non-significant, *t*(19) = 1.18, *p* = .253.

## Discussion

This study aimed to investigate the nature of attentional disengagement for body shape and weight information in those with eating disorders and varying degrees of shape/weight-based self-worth. The findings indicate that, at the later (1300 ms SOA) stage of attentional processing only, different patterns in the allocation of attention were evident between the groups. As hypothesised, the differences in IOR between the control and body images were only observed at the longer SOA, which is to be expected since IOR typically grows as a function of SOA [22].

The attentional results revealed that the High group displayed increased IOR for shape/weight versus control images, indicating a greater level of attentional disengagement, or an avoidance response, in relation to shape/weight images. This was in the opposite direction to the hypothesised effect. It was expected that both the High and ED groups would have difficulty disengaging from disorder-relevant images (i.e., reduced IOR), a finding consistent with research using anxious and depressed populations [23, 24]. The High group instead demonstrated strong attentional disengagement from shape/weight images, which may indicate the potential occurrence of an attentional protective mechanism, as the ongoing processing of such stimuli could serve to exacerbate their shape/weight concerns. Indeed, it may be the presence of this protective cognitive mechanism that has contributed to preventing these at-risk individuals (given their tendency to base their self-worth on shape/weight) from progressing to the levels of eating disorder symptomatology evident in the eating disorder group. This is however a tentative explanation, with replication required to further inform the potential occurrence of this protective cognitive mechanism.

The ED group displayed a similar pattern of results to the High group (i.e., increased inhibition for disorder-salient information relative to non-disorder-salient information), but this did not reach significance. This result may be due to a type II error, or may be indicative of the lack of, or an insufficient, protective mechanism in their attentional processing and hence their higher endorsement of eating disorder pathology. Further, treatment may have had a beneficial role in the reduction of attentional biases, leading to the initial development of such a protective mechanism. One study found that undertaking eating disorder treatment significantly reduced biases towards eating disorder-salient information, when compared with waitlist controls and when controlling for practice effects [42]. Thus the impact of treatment may suggest a growing ability on the part of the ED group to inhibit the ongoing processing of shape/weight stimuli, increasing the potential for this to be a protective factor. Future research using individuals with eating disorders prior to commencing treatment would provide greater clarity regarding the patterns of attentional disengagement for shape/weight information in this population and the potential development of protective mechanisms.

Also contrary to the hypotheses, the Low group displayed decreased attentional disengagement from shape/weight information compared with non-shape/weight information. While unexpected, this pattern of attention is consistent with previous literature demonstrating that human body stimuli are prioritised for attentional selection relative to various types of control stimuli [43]. Importantly, a pattern of decreased disengagement in response to shape/weight images is unlikely to have adverse consequences for these individuals. As these individuals do not heavily invest in shape or weight as a basis for defining their self-worth, any processing of shape/weight images is unlikely to have adverse consequences for their self-worth and, as such, they do not require a protective mechanism.

While increased attentional disengagement from shape/weight stimuli has thus far been conceptualised as a potential protective mechanism, the converse could also be the case in that avoidance could have adverse consequences. The findings of a study that implemented attentional training either towards or away from body shape/weight words in a non-clinical undergraduate population highlight the potential adverse consequences of this pattern of attentional avoidance [44]. The results indicated that those trained to direct their attention away from body shape information subsequently reported greater concerns about their body shape, relative to those trained to direct their attention towards body shape information. Such findings are consistent with the well-established notion that avoidance of threatening stimuli exacerbates and maintains psychological symptoms and distress [45]. Further research is warranted to establish whether an attentional pattern characterised by inhibition of the ongoing processing of shape/weight stimuli elicits harmful or beneficial consequences.

In addition to the limitations and future directions for research already noted, the current study did not include stimuli representing the thin-ideal. Given the lack of clarity regarding attentional processes in relation to thin versus non-thin images, expanding the present research to examine IOR using thin-ideal images is needed. In addition, while the relatively small sample size did not permit such an analysis, further investigation into how these attentional patterns differ across eating disorder subgroups is warranted, given at least some research indicated that attentional patterns may differ across eating disorder diagnoses [46]. Finally, future research would benefit from employing the IOR task in conjunction with an eye-tracking task given that one limitation of the IOR paradigm is its inability to determine whether a decrease in IOR is indicative of reduced time to re-engage with the information or increased time to disengage. The inclusion of eye-tracking would enable researchers to investigate the exact location and duration of participants’ graze duration during the IOR task (Additional file 1).

## Conclusions

The present study was the first to investigate the occurrence and nature of attentional disengagement in a clinical eating disorder sample and those with differing degrees of shape/weight-based self-worth. The current findings indicate that young women without an eating disorder who base their self-worth on shape/weight display a pattern of inhibited processing of shape/weight stimuli in the later stages of attention. The impact of this attentional pattern remains unknown, highlighting the need for further research on the role of attentional avoidance in the development, maintenance, and treatment of eating disorder symptoms.

## Additional file

Additional file 1: Image examples. (DOCX 463 kb)

## Abbreviations

ANOVA: Analysis of Variance
BMI: Body Mass Index
ED: Eating disorder
EDE-Q: Eating Disorder Examination Questionnaire
High: High shape/weight based self-worth group
IOR: Inhibition of Return
Low: Low shape/weight based self-worth group
SOA: Stimulus onset asynchrony

## References

[1] Amerian Psychiatric Association (2013). Diagnostic and statistical manual of mental disorders.

[2] Hargreaves D, Tiggemann M (2002). The role of appearance schematicity in the development of adolescent body dissatisfaction. Cognit Ther Res.

[3] Williamson DA, White MA, York-Crowe E, Stewart TM (2004). Cognitive-behavioral theories of eating disorders. Behav Modif.

[4] Vitousek KB, Hollon SD (1990). The investigation of schematic content and processing in eating disorders. Cognit Ther Res..

[5] Aspen V, Darcy AM, Lock J (2012). A review of attention biases in women with eating disorders. Cogn Emot.

[6] Cisler JM, Koster EH (2010). Mechanisms of attentional biases towards threat in anxiety disorders: an integrative review. Clin Psychol Rev.

[7] Faunce GJ (2002). Eating disorders and attentional bias: a review. Eat Disord.

[8] Dobson KS, Dozois DJ (2004). Attentional biases in eating disorders: a meta-analytic review of Stroop performance. Clin Psychol Rev.

[9] MacLeod CM (1991). Half a century of research on the Stroop effect: an integrative review. Psychol Bull.

[10] Blechert J, Nickert T, Caffier D, Tuschen-Caffier B (2009). Social comparison and its relation to body dissatisfaction in bulimia nervosa: evidence from eye movements. Psychosom Med.

[11] Gao X, Li X, Yang X, Wang Y, Jackson T, Chen H (2013). I can’t stop looking at them: interactive effects of body mass index and weight dissatisfaction on attention towards body shape photographs. Body Image.

[12] Glauert R, Rhodes G, Fink B, Grammer K (2010). Body dissatisfaction and attentional bias to thin bodies. Int J Eat Disord.

[13] Jansen A, Nederkoorn C, Mulkens S (2005). Selective visual attention for ugly and beautiful body parts in eating disorders. Behav Res Ther.

[14] Dondzilo L, Rieger E, Palermo R, Byrne S, Bell J (2016). Association between rumination factors and eating disorder behaviours in young women. Adv Eat Disord.

[15] Roefs A, Jansen A, Moresi S, Willems P, van Grootel S, van der Borgh A (2008). Looking good: BMI, attractiveness bias and visual attention. Appetite.

[16] Glashouwer KA, Jonker NC, Thomassen K, de Jong PJ (2016). Take a look at the bright side: effects of positive body exposure on selective visual attention in women with high body dissatisfaction. Behav Res Ther.

[17] von Wietersheim J, Kunzl F, Hoffmann H, Glaub J, Rottler E, Traue HC (2012). Selective attention of patients with anorexia nervosa while looking at pictures of their own body and the bodies of others: an exploratory study. Psychosom Med.

[18] Most SB, Simons DJ, Folk CL, Gibson BS (2001). Attention capture, orienting, and awareness. Attraction, distraction and action: multiple perspectives on attentional capture.

[19] Fairburn CG, Cooper Z, Shafran R (2003). Cognitive behaviour therapy for eating disorders: a “transdiagnostic” theory and treatment. Behav Res Ther.

[20] Williamson DA, Muller SL, Reas DL, Thaw JM (1999). Cognitive bias in eating disorders: implications for theory and treatment. Behav Modif.

[21] Posner MI, Cohen Y, Bouwhuis HBDG (1984). Components of visual orientating. Attention and performance X: control of language processes.

[22] Klein RM (2000). Inhibition of return. Trends Cogn Sci.

[23] Perez-Duenas C, Acosta A, Lupianez J (2009). Attentional capture and trait anxiety: evidence from inhibition of return. J Anxiety Disord.

[24] Dai Q, Feng Z (2009). Deficient inhibition of return for emotional faces in depression. Prog Neuro-Psychopharmacol Biol Psychiatry.

[25] Carters MA, Rieger E, Bell J (2015). Reduced inhibition of return to food images in obese individuals. PLoS One.

[26] Janelle CM, Hausenblas HA, Ellis R, Coombes SA, Duley AR (2009). The time course of attentional allocation while women high and low in body dissatisfaction view self and model physiques. Psychol Health.

[27] Geller J, Johnston C, Madsen K (1997). The role of shape and weight in self-concept: the shape and weight based self-esteem inventory. Cognit Ther Res..

[28] Geller J, Johnston C, Madsen K, Goldner EM, Remick RA, Birmingham CL (1998). Shape-and weight-based self-esteem and the eating disorders. Int J Eat Disord..

[29] Nijs IM, Franken IH (2012). Attentional processing of food cues in overweight and obese individuals. Curr Obes Rep.

[30] Fairburn C, Beglin S, Fairburn C (2008). Eating disorder examination questionnaire. Cognitive behavior therapy and eating disorders.

[31] Australia Beauro of Statistics: Profiles of Health, Australia, 2011–2013 (catalogue no. 4338.0). Canberra, Australia: Australian Bureau of Statistics; 2012.

[32] Bradley MM, Lang PJ, Coan A, Allen JJB (2007). The international affective picture system (IAPS) in the study of emotion and attention. Handbook of emotion elicitation and assessment.

[33] Fairburn CG. Cognitive behavior therapy and eating disorders. New York: Guilford Press; 2008.

[34] Fairburn CG, Cooper Z, Fairburn CG, Wilson GT (1993). The eating disorder examination (12th ed.). Binge eating: nature, assessment and treatment.

[35] Mond JM, Hay PJ, Rodgers B, Owen C, Beumont PJ (2004). Validity of the eating disorder examination questionnaire (EDE-Q) in screening for eating disorders in community samples. Behav Res Ther.

[36] Aardoom JJ, Dingemans AE, Op't Landt MCS, Van Furth EF (2012). Norms and discriminative validity of the eating disorder examination questionnaire (EDE-Q). Eat Behav.

[37] Luce KH, Crowther JH (1999). The reliability of the eating disorder examination-self-report questionnaire version (EDE-Q). Int J Eat Disord..

[38] Rose JS, Vaewsorn A, Rosselli-Navarra F, Wilson GT (2013). Test-retest reliability of the eating disorder examination-questionnaire (EDE-Q) in a college sample. J Eat Disord.

[39] Mond JM, Hay PJ, Rodgers B, Owen C, Beumont PJ (2004). Temporal stability of the eating disorder examination questionnaire. Int J Eat Disord..

[40] Koelstra S, Muhl C, Soleymani M, Lee J-S, Yazdani A, Ebrahimi T, Pun T, Nijholt A, Patras I (2012). Deap: a database for emotion analysis; using physiological signals. IEEE Trans Affect Comput.

[41] Stoyanova RS, Pratt J, Anderson AK (2007). Inhibition of return to social signals of fear. Emotion.

[42] Shafran R, Lee M, Cooper Z, Palmer RL, Fairburn CG (2008). Effect of psychological treatment on attentional bias in eating disorders. Int J Eat Disord..

[43] Downing PE, Bray D, Rogers J, Childs C (2004). Bodies capture attention when nothing is expected. Cognition.

[44] Engel SG, Robinson MD, Wonderlich SJ, Meier BP, Wonderlich SA, Crosby RD, Steffen KJ, Mitchell JE (2006). Does the avoidance of body and shape concerns reinforce eating disordered attitudes? Evidence from a manipulation study. Eat Behav.

[45] Kashdan TB, Barrios V, Forsyth JP, Steger MF (2006). Experiential avoidance as a generalized psychological vulnerability: comparisons with coping and emotion regulation strategies. Behav Res Ther.

[46] Giel KE, Teufel M, Friederich HC, Hautzinger M, Enck P, Zipfel S (2011). Processing of pictorial food stimuli in patients with eating disorders—a systematic review. Int J Eat Disord..

