# Supplementary figures and images for: Inhibition of return for body images in individuals with shape/weight based self-worth
Source: J Eat Disord. 2018 Sep 14;6:25. doi: 10.1186/s40337-018-0211-5 (PMC6137877; doi:10.1186/s40337-018-0211-5)

**Image Examples**

Example of Body Shape and Weight Images


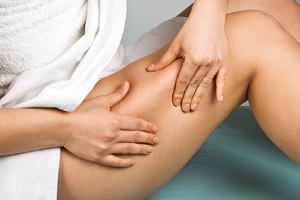


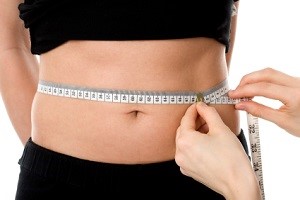


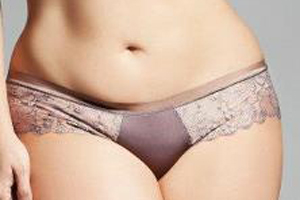


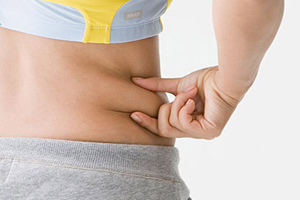


Example of Animal Images


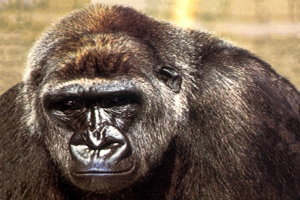


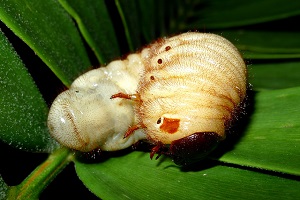


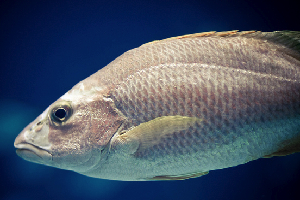


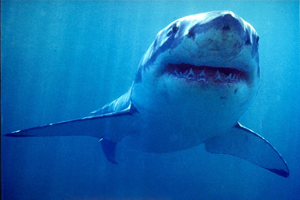

Supplement: Supplementary file 1 — Image examples. (DOCX 463 kb) [file 40337_2018_211_MOESM1_ESM.docx]
